# Preliminary Communications Regarding an Enquiry into Artificial Digestion

**Published:** 1838-07-01

**Authors:** 


### PRELIMINARY COMMUNICATIONS REGARDING AN ENQUIRY INTO ARTIFICIAL DIGESTION. By Prof. Dr. *Purkinje* and Dr. *Pappenheim*, in Breslau.

### I. ON THE INFLUENCE OF GALVANISM IN ARTIFICIAL DIGESTION.

(From the Archiv Für Anatomie, Physiologie, von Dr. J. Müller, 1838.)

WHEN, in the May Number of last year, we just commenced some preliminary experiments on artificial digestion of the mucous membrane of the stomach, in hydrochloric acid, at "brood-heat*;" and when the residua of muscles, nerves, and nervous fibres given up to digestion, were examined by us under the microscope, we were struck every time at finding a great quantity of oblong, knotty, conferve-shaped bodies in company with those organic substances, which at first we could not ascribe to the mucous membrane of the stomach, because we supposed that this, according to the common theory, is a simple mucous membrane only furnished with some scattered glands. It is therefore not to be wondered at that the idea forced itself on us to take these conferve-shaped bodies for a new organic product of the digestive process, as the commencement of a new organization. This however was soon explained, when the mucous membrane of the stomach† was subjected to a strict microscopic examination. For it appeared at once, that those bodies are nothing else but the parenchyma of extremely small, long, simple glands, which constitute the chief part of that mucous membrane. After carefully separating the innermost tolerably thick layer of the mucous membrane of the stomach of the ruminantia, or of the simple stomach of other animals, entirely from the subjacent cellular layer, a thing which may be effected in very many parts over which it lies in folds, except the pyloric portion (at which it is firmly united with the middle coat) in large patches frequently several inches square, it was found on closer examination, that this apparently simple mucous membrane is composed of an immense number of very small,

---

* The reader will excuse the coinage here attempted. We have no term in the language to express the *brut-wärm* of the German, by which is meant the temperature of incubation. Certainly an Englishman is as well entitled to coin the term *brood-heat*, as a German is to make the term *brut-wärm*. Besides we have analogy for our new term; we have the term *brood mare.—Rev.*

† The term in the original is laab-magen, by which is meant that stomach of ruminating animals in which the runnet is found.

oblong, cylindrical, simple glands, which are imbedded in a perpendicular direction in the surrounding cellular and vascular tissue. This structure may be very clearly perceived by allowing these separated patches of mucous membrane to harden in a concentrated solution of carbonate of potash from 12 to 24 hours, in a gentle heat, where it may then be divided in all directions into very fine, transparent slices, which, when brought under the microscope, show the internal structure of that membrane.

From this investigation it follows that all this apparently mucous layer is glandular throughout, and may be considered as a glandular membrane. The more minute description of its structure in man or other animals is reserved for another place.

If such a portion of the glandular membrane be combined with the proportionate quantity of water and muriatic acid, so as to constitute the digestive fluid, the digestive action is next directed to the solution of the cellular and vascular tissue which holds these glands together, and the glands, which themselves remain undissolved, swim in great numbers in the digestive fluid singly and uncombined, in the form of those knotty bodies. In this simple glandular substance filled with parenchymatous grains the peculiar runnet-material *(Laabstoff)* seems to be contained, on which all the phenomena of digestion in connexion with the other conditions are dependant. Besides those glands constituting the gastric mucous membrane, there are also found separate, scattered, larger glands, which seem to be destined for the secretion of a simple mucus, whilst those glands by virtue of their peculiar glandular substance were destined for the exclusive secretion of the digestive or gastric juice which proceeds immediately from their substance.

After we supposed that in this way we had found the proper digestive principle in this glandular substance, the question arose whether it is of itself sufficient to effect artificial digestion. After the glandular membrane of the stomach was detached as cleanly as possible, it was brought into the brood-heat, partly in the recent state, in various series of quantities, with a constant quantity of distilled water, and with small portions of hard-boiled albumen, in order to observe the action exercised on this latter substance. It was soon discovered that after three hours or more the changes accompanying digestion were not observable in the albumen ; on the contrary after from 8 to 12 hours, a very rapid putrid fermentation took place in which it terminated on continuing the experiment for a longer time. Thus the mere gastric substance was found to be insufficient of itself to effect the digestive process This, it is known, may be accomplished by the addition of small quantities of acid, as for instance, by means of hydrochloric acid. The question now arose by what organic process the hydrochloric acid is set free in the living stomach, and what other acid comes into play during digestion. Our microtomical investigation certainly discovered no separate organ in the stomach, except probably those simple mucous glands, which could secrete the acid required for digestion ; it must therefore be those *gastric glands* that immediately take on them the secretion of the digestive mucus and of the acids at the same time. The thought now presented itself whether some process of the nerves similar to that of galvanism may not effectuate this secretion in the stomach. The separation of the acids should then be accomplished, through such dynamic influence, either in the alimentary substances, or in the intermixed saliva and mucus, or in the serum of the blood contained in the blood-vessels of the gastric mucous membrane, or finally in the gastric substance itself. This observation gave rise to the following series of experiments :—

When in the month of August we set about repeating these experiments with a pile consisting of 30 pairs of plates 4 inches square, in which platina wires were brought from both poles into one and the same glass vessel, no result whatever appeared; where pure gastric membrane came into the experiment putrefaction rapidly took place in brood-heat. The hard-boiled albumen was then either unchanged, or had become dirty and discoloured, or it was, on the application of muriate of soda, considerably hardened. Only once, when wrapped up in a bit of cloth, at the acid pole, it was introduced with pure gastric substance into distilled water, we found that after 12 hours it had disappeared from the cloth, and that it was dissolved in the fluid. The experiments were now changed thus far, that in order to separate the action of the poles, and to produce at the one the development of a pure acid, two glasses were selected, into each of which the mixtures used for the investigation were introduced, and which were connected by a cotton thread moistened with water.

A. In order to ascertain the possible action of the substances entering into the stomach from without under the influence of a dynamic agent, the experiment was first made with saliva, as this even in the natural state of things generally evinces an acid reaction. Saliva was put into two glasses to the amount of two drachms, both were exposed to the action of the galvanic poles, and they were connected together by means of a moist cotton thread. At the acid pole there was a development of chlorine gas quite sensible to the smell. After 24 hours the acid reaction was already so strong, that the saliva had a perceptibly acid taste, and litmus paper was intensely reddened by it. The re-action at the alkaline pole we leave out of consideration, it not being necessary to the purpose of our experiments. By chemical re-agents the acid developed was proved to be hydrochloric acid. Into the acid saliva thus obtained three grains of dried gastric membrane were put, and then three square bits of dried albumen, and the mixture was exposed to brood-heat. In the ordinary time of digestion no alteration was as yet perceptible in the albumen. The fluid however very perceptibly smelt of hydrochloric acid, and subsequently it developed the ordinary acid smell of the digestive fluid. The albumen was not discoloured, nor opaque or of a chalk-white appearance, but it presented its ordinary transparency, a circumstance which left us to expect a possible farther change. Not till after the lapse of 8 hours did we perceive the characteristic transparent edges of the albumen passing into the digestive fluid. The mixture was now again placed in brood-heat, and when examined on the following morning, that is after the lapse of 22 hours, the digestion-edges (verdauungsränder) were advanced to be sure towards the middle, but the angles were not rounded, and in several places the mass was split and almost softened to a jelly. From this experiment it therefore followed, that if a dynamic action, similar to galvanism, takes place in the stomach, the saliva introduced with the alimentary substances is still capable of setting free a portion of the hydrochloric acid necessary for digestion.

B. In the next place it occurred to us to institute a similar experiment with a dilute solution of chloride of sodium, which enters the stomach partly as an immediate constituent, partly as an artificial admixture with the food. As the result however might here be anticipated with certainty, this experiment was not instituted, and the possibility of a similar production of an acid from chloride of sodium, as well as from saliva, was immediately pre-supposed.

C. *Experiment with diluted Albumen.*—Into each glass which was again to be exposed to the action of the poles, three drachms of distilled water with about one drachm of albumen, well mixed together, were put, upon which the action of the galvanic poles displayed itself in the following manner: at the oxygen pole, coagulated albumen collected around the platinum wire in large flocculi, and the fluid in the vicinity of the wire evinced an acid re-action. In the other glass, at the hydrogen pole, the fluid was on the contrary observed to present no appearance of coagulation, being uniformly diluted and thinner than at the commencement. It had a weakly alkaline re-action. After twenty-four hours the alkalinity was very considerable at the latter pole; on the contrary the acid re-action continued at the other, only always in a weak degree, probably because the acid was partly expended in coagulating the albumen. When a portion of this acid fluid was filtered and then tested, it showed evident traces of hydrochloric acid.

D. *Experiments with Mucus.*—Four drachms of nasal mucus mixed no doubt with some saliva were collected, the saliva was then diluted with distilled water, as much as possible, and separated from the mucus. The mucus thus purified, was rubbed up in a stone mortar with about one-half the quantity of distilled water, and about three drachms were then introduced into the glasses which were to be subjected to the galvanic action. The mucus presented the same phenomena as the albumen. At the acid pole flakes of mucus collected around the platinum wire, with several air-bubbles, with a slight acid re-action, so that the wire, from time to time required to be freed from the attached mucus, in order that the other parts of the fluid also, which continued to develope chlorine gas sensible to the smell, might be exposed to the galvanic action. After twenty-four hours the entire fluid presented a tolerably strong acid re-action. Here also the presence of free hydrochloric acid was ascertained. At the alkaline pole the mucous solution was found to be thinner, and gave an alkaline re-action.

E. *Experiments with the Constituents of the Blood.*—It is commonly admitted that on many organic surfaces, as on the inner surface of the uterus, on serous membranes, and on the parts of mucous membranes not furnished with glands, the serous constituent of the blood is separated immediately from this fluid. We do not mean to enquire here how far this admission is correct; it sufficed, however, to suggest to us the question, whether acid separated by this supposed dynamic action immediately on the serum of the blood, and secreted on the surface of the stomach, might not serve as a condition of digestion. Pure human serum, was put into both glass vessels in the same way as has already been mentioned of the saliva. After twenty-four hours the fluid was sufficiently acid at the oxygen pole; the acid re-acted in the same way as the hydrochloric acid. Whether other acids had been developed, was not enquired into; this appeared unnecessary, as other acids, in small quantity, would not have so acted as to prevent digestion. Into two drachms of this fluid the usual quantity of three grains of gastric membrane with three squares of albumen were put in the brood-heat. After a few hours the albumen appeared yellow and discoloured, the edges opaque. The gastric substance was dissolved to be sure, exhibited the well-known, acid (brodsauren) smell, and no trace whatever of putrefaction, which was probably hindered by the sufficient acidity of the fluid; but the albumen even after twenty-four hours did not undergo the ordinary digestive changes, at the very most it was softened, and discoloured. As a parallel-experiment another glass with two drachms of distilled water, four grains and a half of gastric substance and three drops of hydrochloric acid, with just as much hardened albumen, as before, was prepared, and into this two drachms of serum were put; the entire was placed in brood-warmth. Here the added hydrochloric acid was to replace the acid developed in the galvanic process, and our object is to see whether the mixture of the serum did not exercise a preventive effect on the digestion. After the same interval the same change in the albumen manifested itself, whence accordingly the preventive action of the serum appeared to proceed. It would also appear manifest from this experiment that this presumed secretion and dynamic acidifying property of the serum of the blood cannot yield the acid principle required in natural digestion. This experiment is altogether imperfect, and rests on a basis not at all sufficiently grounded. Nature might still have means innumerable to produce in the serum of the blood that change which should be directly necessary for the process of digestion. It lay, however, in the regular course of our enquiries, and it should not be passed over, though it was to yield but a negative result.

Of the same quality is the following experiment, which was instituted with as concentrated a solution as possible of the red portion of the blood. After a piece of blood-cake was previously washed in distilled water so as to free it of any serum that may be attached to it, it was cut up into small portions, and distilled water was poured over it. Of the solution of the red part of the blood thus obtained, the due quantities, in the same way as has been already mentioned, were exposed to the action of galvanic electricity, and thus an acid fluid was obtained at the oxygen pole. With respect to the contents of hydrochloric acid we still remained doubtful. Experiments with fibrine were not undertaken.

F. *Experiments with the Substance of the Gastric Mucous Membrane* (Laab).—The chief question now to be determined was, whether the proper organic substance of the gastric mucous membrane itself does not contain already prepared the materials, which under the dynamic influence of the nerves set free the acid necessary to digestion. Accordingly some dried gastric mucous membrane of an ox at the temperature of + 18° R. was finely powdered, and about three grains with two drachms of distilled water, both put into a glass, were exposed to the action of the galvanic pile. The fluid in the one glass soon evinced an acid, whilst that in the other glass showed an alkaline re-action. In the former chlorine gas was evolved.

Into each of the glasses a piece of albumen was introduced at the same time. In this experiment after the lapse of sixteen hours, the edges of the piece of albumen were observed to be transparent in the acid fluid. After eighteen hours the albumen was in a great measure dissolved. The acid fluid re-acted as hydrochloric acid, in the alkaline fluid the albumen was to all appearance unchanged. This experiment convinced us that the substance of the gastric mucous membrane, when placed under galvanic influence, is capable of yielding the quantity sufficient for setting free the acid in the artificial process of digestion: An attempt was now made to obtain the artificial digestive fluid from the mere gastric membrane without the help of acid. Several drachms of acid digestive fluid were prepared under the influence of the galvanic pile, and one portion of it, with albumen, was given up to digestion by itself in brood-heat; the other portion was likewise put into the albumen, and also in the brood-heat, and remained in connexion with the galvanic pile. In the former portion, after the lapse of three hours the edges of the piece of albumen were observed to be transparent, whilst in the other the piece was almost entirely dissolved.

From this it appeared that the acid developed in the gastric membrane is of itself sufficient perfectly to accomplish digestion; but with the help of galvanism the development of the acid is continued, and so digestion follows still more rapidly. It is to be understood that in the latter experiment, whilst the acid pole was introduced into the digesting mixture, the alkaline pole had been at the same time introduced into a similar digesting mixture with a connecting moistened cotton thread. The question now arises—did the digestion follow here more rapidly in consequence of the albumen having been exposed at the same time to the influence of the oxygen pole, and so a greater disposition to solution was in some measure developed in it, or did it follow in consequence of a greater quantity of developed acid from the gastric substance? It might here become the subject of enquiry in what proportion the acid was contained in the first digestive fluid, where the digestion proceeded more tediously, in which case the more tedious progress might be explained from the too small quantity, on which point we require another series of experiments to inform us. It might farther become the subject of enquiry whether so much acid would be developed from the gastric membrane through mere galvanic influence that it would suffice by its excess to prevent digestion; then another series of experiments would be necessary to inform us under what conditions hard-boiled albumen exposed to the oxygen pole could be made more or less soluble in the ordinary mixture. These experiments however do not belong immediately to the present enquiry. It sufficed for the present to have ascertained that the gastric mucous surface itself contains within it sufficient materials to set free the necessary digesting acid under galvanic influence. The question now again arose, whether chlorine salts soluble in water were incidental to the gastric membrane, or were admixed through other secretions developed by this hydrochloric acid under the action of galvanism. It further remained as a subject of enquiry whether the gastric mucous surface free from chlorine salts was still capable, under the influence of galvanism, to set free the quantity of acid necessary to digestion. In this case the chlorine must be contained in an elementary form in the gastric mucous membrane, from which the acid should be formed again by combining with hydrogen at the galvanic pole. This however may be reserved for further investigation.

From the experiments thus far carried the following results might be deduced:—

1. It is unnecessary to admit a peculiar secreting organ for the hydrochloric acid in the stomach, because, with the exception of the secreting glands of the gastric membrane and the mucus, no separate organ is found for that purpose, as follows from our microtomical enquiry, as must happen for so specific a substance as hydrochloric acid is, and would scarcely be explicable on the hypothesis of mere transudation, as in the case of serum.

2. From our galvanic experiments it follows that the juices mixed with the food in the natural way, the saliva, the mucus, the portions of chloride of sodium and albumen generally present therein, further the serum of the blood which is possibly mixed in the stomach by exudation, but most of all the gastric mucous substance itself, develope as much chloride of sodium, as is required for the digestion of the coagulated albumen.

3. Were the nervous action in the stomach either identical with that of galvanism, or acting in a manner analogous to it, or at least accompanied by a galvanic process, this would seem to be sufficient to account for the development of the hydrochloric acid necessary for digestion, without our being obliged to admit a separate act of secretion for the purpose.

It might now be asked whether this enquiry could be brought to a perfect solution in the way of vivisection by applying galvanism to the nerves which preside over digestion; by this process also the analogy between galvanism and nervous action could either be fully established, or rendered still more doubtful; the further investigation of this matter, however, we defer for a more favorable opportunity.

### II. On the Effects of certain Mechanical Agents in the artificial Digestion of coagulated Albumen.

A. Our next object then was to imitate the agents existing in nature which act on the food. To this head belongs first of all the division of the food by the teeth. Three grains of hard-boiled albumen were divided into fragments of about $\frac{1}{2}'''$ in diameter, and together with the ordinary mixture of artificial digestive fluid (gastric substance, gr. ij., water, dr. ij., concentrated hydrochloric acid, gutt. ij.) were put into a vessel, and for the sake of comparison, three large pieces of albumen, together of the same weight, (three grains) were put into another vessel in a similar mixture, at the temperature of brood-heat: it appeared that after one hour and half the solution was completely finished in the first vessel, whilst in the other vessel even after four hours traces of undissolved albumen were still observable. It seems unnecessary to say more on the further application of this experiment to the ordinary physiological processes.

B. It is usually supposed that, by the contractions of the several muscular fibres of the stomach, the alimentary materials are brought into new contact partly with the surface of the mucous membrane, and partly with the solvent juices of digestion, whereby the changes and solution of the same must go on more rapidly, than if, the fluid remaining in a state of rest, the surrounding saturated menstruum prevented or retarded the approach of new substances for solution. It was according to the purpose to institute an analogous experiment with the fluid of digestion and albumen, by placing them in constant motion, and noting the time of complete solution. For this experiment we selected a double vessel of tin. Into the space between the external and internal vessel water at the temperature + 30 R. was poured, and it was corked up; into the internal space two ounces of digestion-fluid with 48 grains of albumen were put. By means of water renewed from time to time, the temperature of the inner reservoir was constantly kept up at 28° R. The entire reservoir was shaken for about 2½ hours, and then presented all the albumen dissolved. In the absence of motion, all other circumstances remaining the same, the solution of the same quantity of albumen follows in about three hours, as we may infer from former experiments.

c. The stomach, more or less filled with alimentary substances, and the digestion-fluid presses partly with its muscular tissue equally on all sides on its contents, whilst this pressure, during respiration, is performed still further removed from the pressure of the atmosphere by the surrounding muscular parietes of the diaphragm and abdomen. It is to be admitted that by such pressure the solid constituents are much more intimately penetrated by the fluids, and are accordingly more acted on than without the same. In order to find a new datum of analogy this calculation gave occasion to the following experiment:—

To a glass of nearly four cubic inches a barometer-tube 28″ in length, 1½ in diameter, was fitted hermetically, and six drachms of digestion-fluid with 20 grains of albumen were added, so that the barometer-tube was filled with the same fluid to the stated height.

The pressure of the entire pillar of fluid amounted to 4½ pd. In this case the perfect solution followed in 2½ hours, accordingly much more rapidly than under ordinary pressure. A still earlier, but less exact experiment, in which the albumen contained in a cloth was exposed to slight pressure in the fluid, gave a similar result.

From all this it follows that, in order to conclude from analogy, the pressure of the stomach and of the abdominal parietes on the alimentary materials existing in the digestion-fluid, must form a very important element of the reasoning.

